# Investigating intentionality in elephant gestural communication

**DOI:** 10.1098/rsos.242203

**Published:** 2025-07-09

**Authors:** Vesta Eleuteri, Lucy Bates, Yvonne Nyaradzo Masarira, Joshua M. Plotnik, Catherine Hobaiter, Angela S. Stoeger

**Affiliations:** ^1^ Department of Behavioral and Cognitive Biology, University of Vienna, Vienna, Austria; ^2^ School of Psychology, Sport and Health Sciences, University of Portsmouth, Portsmouth, UK; ^3^ Elephant CREW, Victoria Falls, Zimbabwe; ^4^ Department of Psychology, Hunter College, City University of New York, New York, NY, USA; ^5^ Department of Psychology, The Graduate Center, City University of New York, New York, NY, USA; ^6^ School of Psychology and Neuroscience, University of St Andrews, St Andrews, UK; ^7^ Acoustic Research Institute, Austrian Academy of Sciences, Vienna, Austria

**Keywords:** elephant, gestural communication, elephant gesture, language, intentional communication

## Abstract

A crucial feature of language is the ability to communicate cognitive goals to a specific audience, i.e. goal-directed intentionality. Core criteria for this ability include (i) audience directedness: signalling in the presence of an attentive audience, (ii) persistence: continuing signalling until goals are met, and (iii) elaboration: using new signals following communicative failure. While intentional use has been demonstrated in individual gestures in some non-primates, primates—in particular apes—show this ability across many gestures. But is goal-directed intentionality across many gestures restricted to primates? We explored whether savannah elephants use many gestures with goal-directed intentionality. We presented semi-captive elephants with desired and non-desired items, recording their communicative attempts when an experimenter met, partially met or failed to meet their goal of getting the desired item. Elephants used 38 gesture types almost exclusively when a visually attentive experimenter was present, demonstrating audience directedness. They persisted in gesturing more when their goal was partially as compared with fully met but showed no difference in persistence when the goal was met or not met. Elephants elaborated their gesturing when their goal was not met. We find goal-directed intentionality across many elephant gestures and reveal that elephants, like apes, assess the communicative effectiveness of their gesturing.

## 1. Background

One ability is often considered to distinguish humans from other beings: language [1]. But what makes human language unique? In other species, communicative signals enable the transmission of rich information, and recipients respond to such information, using it to guide their behaviour [2]. Language, however, is different. With language, we do more than broadcast information. We use language *intentionally* to flexibly communicate a range of cognitive goals (i.e. meanings) to a partner while taking their mental states into account [3,4]. When we fail, we persist—and may elaborate—our communicative attempts to better convey our intended meaning. Intentionality has been distinguished into different ranked categories (termed orders; [3]). Zero-order intentionality is attributed to signals produced as reactions to stimuli, with no intention of communicating a goal (e.g. a yelp in pain in response to picking up hot coal off the fire). Goal-directed (or first-order) intentionality is attributed to signals produced with the intention of communicating a cognitive goal that modifies a recipient’s behaviour (e.g. ‘stop there’ if the recipient is approaching the hot coals). Second-order intentionality is ascribed to signals produced with the intention of changing a recipient’s mental state, such as their knowledge or understanding (e.g. when we inform a recipient that the coals are still hot). We can see the progressive development of these orders of intentional communication in human infants, here termed as ‘illocutionary’ (first-order) and ‘perlocutionary’ (second-order) acts [5]. Other orders of intentionality are possible: for example, ‘Fred knows that Frank intends for Freya to think that Flora knew all along’ is fourth-order intentional. Given that first-order intentionality requires a cognitive intention about another individual’s behaviour and second-order intentionality (and above) an intention about another individual’s mind, these two stages are typically considered to distinguish human language from other species’ communication and have been used as benchmarks of language development in ontogeny [5,6].

To establish goal-directed intentionality of at least the first order, one or more of the following intentionality criteria needs to be regularly met [5,7–12]: (i) audience directedness—signallers use signals in the presence of an audience and select their modality (e.g. visual, tactile) appropriately according to the audience’s state of visual attention; (ii) persistence—signallers persist signalling when their goal is not fully met; or (iii) elaboration—signallers change signals when their initial signals fail to meet their goal.

Non-human apes (hereafter apes) and—to a more limited extent—a few other primates are known to regularly communicate with goal-directed intentionality across many gesture types [7,13–16]. A seminal experiment by Leavens *et al*. [8] revealed that captive chimpanzees gesture with goal-directed intentionality, finding persistence and elaboration when they failed or partially failed at meeting their goal. While there is no evidence that apes gesture with second-order intentionality to change their recipient’s mental states, there are indications that apes possess some knowledge of what others know [17,18] and that they adjust their gesturing based on their recipient’s understanding [7]. Specifically, Cartmill & Byrne [19] applied Leavens *et al.*’s experimental design [8] to captive orangutans, showing persistence when their goal was not met and the use of different communicative strategies (i.e. gesture repetition versus elaboration) when a human experimenter completely failed to meet or only partially met their goal of getting a preferred food. Specifically, orangutans repeated the previous (partially successful) gesture types after the experimenter partially met their goal (i.e. gave them half of the preferred food) but elaborated by using different gesture types after the experimenter completely failed to meet their goal (i.e. gave them the non-preferred food). The use of different communicative strategies suggested that orangutans take into account the communicative effectiveness of their gestures in achieving their goal and that they may consider the experimenter’s understanding when gesturing (second-order intentionality). When the experimenter appeared to understand them by providing the desired food, they repeated the same gesture types. In contrast, when the experimenter appeared to misunderstand them by offering the non-desired food, they switched to different gesture types.

But is intentional gesturing across many signal types restricted to apes? We are closely related to the other ape species, and we share with them a similar body plan and some social goals, which limits our ability to determine whether commonalities in our communication are due to common ancestry, similar anatomy or shared socio-cognitive pressures. To understand which of these factors may have driven the evolution of intentional communication, we need to look beyond primate taxa to species that are both more distantly related and anatomically different from us.

Beyond primates, goal-directed intentional gesturing has been shown in several species, from a fish to different bird species, but—so far—this capacity has only been shown in one or two gesture types used for highly specific purposes [20–23]. For example, coral reef fish produce a referential gesture to indicate prey when cooperatively hunting, while Arabian babblers use object presentation and babbler walk to initiate joint travel [20]. Some species produce ritualized displays that may also appear to meet the criteria for intentional use [24–27]. For example, several bird species are known to produce courtship displays towards visually attentive females (apparently meeting audience directedness) that involve sequences of actions that continue until the female mates or leaves (apparently meeting persistence) [24,26]. However, direct tests of the criteria for intentionality are needed to establish the goal-directed intentional use of displays in birds and other animals. In addition, where a signalling behaviour (e.g. courtship display) is phylogenetically ritualized for a specific purpose (e.g. mating in birds) and, thus, typically consists of fixed sequences of actions under strong selective pressure, it is challenging to establish the use of different signals and their flexible use across contexts and goals that is characteristic of intentional language-like communication. In such ritualized behaviour, mechanistic explanations may be more parsimonious than ascribing a mental state (e.g. intended goal) to the signaller [3,12,28]. For example, arousal may explain the persistent use of signalling in a courtship display [9]. In contrast, mechanistic explanations become less plausible for the use of very large sets of signals and when signals are deployed with substantial flexibility across contexts and goals within a species [3,12,28]. Wild apes employ large repertoires of over 100 gesture types [29,30] that meet multiple intentionality criteria, and they use these gestures to flexibly communicate a range of meanings (i.e. goals) across a variety of contexts to different types of recipients throughout the day [7,30–35]. To date, no other non-human species has been shown to demonstrate similar levels of systematic, flexible intentional use across large sets of gesture types. In contrast, evidence for intentional use in ape vocalizations remains scarce [36,37] and is limited to a single alarm call [38–40]. The current lack of evidence in the ape vocal domain may be due, in part, to the challenge of determining audience directedness and attention in the auditory domain (see [9] for detailed discussion). For example, vocalizations are potentially heard by many individuals, including where they are out of sight, making it challenging to establish who the recipient is. As a result, here we focus on exploring intentionality in the gestural domain.

Elephants are physically different and evolutionarily distant from us. But, like us, they are long-lived and large-brained animals, show remarkable cognitive capacities in their social behaviour and live in a multi-level, fission–fusion social structure where intentional communication may be advantageous in regulating social dynamics. Elephants have been described to use many visual and tactile body acts across different behavioural contexts, suggesting vision and touch are important for social communication [41–43]. A study by Smet & Byrne [44] showed audience directedness in semi-captive elephants, who were able to target visual gestures towards a human experimenter when that experimenter was present and according to her state of visual attention. We recently demonstrated that this ability extends to greeting gestures between semi-captive elephants: a first step towards demonstrating goal-directed intentionality in conspecific elephant gesturing [45]. When greeting each other, elephants appropriately targeted the sensory modality of their gestures given their recipients’ ability to perceive the gestures. However, because greeting does not involve the signaller requesting a specific behavioural reaction (a goal), it remained unclear whether elephants show goal-directed persistence and elaboration in their gesturing, both key criteria in assessing the presence of intentional communication [8,12,28].

Here, we explore intentionality in elephant gestures by testing for audience directedness, persistence and elaboration, building on the experimental paradigm previously employed to first test for intentional gesturing in captive chimpanzees and orangutans [8,19]. We presented semi-captive elephants with out-of-reach desirable and non-desirable items, allowing them the opportunity to gesture to a human experimenter to request the desired item (their goal). We created three reaction situations in which the elephants’ goal would be fully met, partially met or not met. We predicted that, if elephants communicate with a goal in mind, they should (i) direct their gestures towards the experimenter or the desired item [8], (ii) use gestures only in the presence of a visually attentive experimenter (i.e. audience directedness), and (iii) persist in further gesturing when their goal was not met or only partially met, as compared with when it was fully met (i.e. persistence) [8]. In addition, if elephants evaluate the communicative effectiveness of their gestures at achieving their goals, they should (iv) use novel gestures rather than repeat previous ineffective ones when their goal was not met, as compared with when it was fully met or partially met (i.e. elaboration; following [19]). Lastly, (v) elephants should use more gesture types (i.e. diversity) when their goal was not met as compared with when it was fully or partially met, in order to increase the likelihood of producing gestures effective at achieving their goal.

## 2. Material and methods

### 2.1. Study site and subjects

Experiments were conducted between March and May 2024 in the Victoria Falls area in Zimbabwe. Subjects were 17 semi-captive African savannah elephants (*Loxodonta africana*) living in one group in the Jafuta Reserve (eleCREW; −18.020542, 25.804887) and in two separate groups in the Victoria Falls National Park (WildHorizons1; WildHorizons2; −17.968185, 25.837943). Subjects were eight adult males and nine adult females. Following Poole & Granli [43], we grouped subjects into two age categories according to whether they were younger or older than 35 years old. We defined ‘young adults’ as subjects between 15 and 34 years old and ‘old adults’ as subjects at least 35 years old (table 1). The elephants roam free in their habitat during the day and stay together in stables at night. They are engaged in non-invasive interactions with tourists and locals for which they receive positively reinforced behavioural training on a daily basis. Specifically, in the early morning, the elephants receive a 15–30 min training session with handlers where they are taught to perform specific behaviours (e.g. give object) by being rewarded with pellets and during which they are kept tethered temporarily for safety reasons. Two other female elephants from eleCREW showed no attempts at communication with the experimenter across trials and were thus not included in the study.

**Table 1.** Subjects with demographic information.

subject	group	sex	age group
Doma	eleCREW	male	old adult
Mainos	eleCREW	male	young adult
Detema	eleCREW	male	young adult
Laduma	eleCREW	male	young adult
Moka	eleCREW	male	old adult
Hwange	eleCREW	female	young adult
Tatu	eleCREW	female	old adult
Jock	WildHorizons1	male	old adult
Pfumo	WildHorizons1	male	young adult
Emely	WildHorizons1	female	young adult
Naledi	WildHorizons1	female	young adult
Jenet	WildHorizons1	female	young adult
Jambo	WildHorizons2	male	old adult
Tandihwe	WildHorizons2	female	young adult
Ntombe	WildHorizons2	female	young adult
Coco	WildHorizons2	female	old adult
Tendai	WildHorizons2	female	young adult

### 2.2. Experimental design

Subjects were presented with a desired item and a non-desired item. The desired item consisted of a tray containing six apples, while the non-desired item was an empty tray. The food type was chosen after consulting with handlers about the elephants’ food preferences. Each subject was presented with three experimental trials corresponding to three conditions:

— *Goal met condition* (i.e. successful communication). All available apples were delivered from the tray.
— *Goal not met condition* (i.e. unsuccessful communication). Empty tray was delivered.
— *Goal partially met condition* (i.e. partially successful communication). One apple was delivered from the tray.

**Figure 1.**
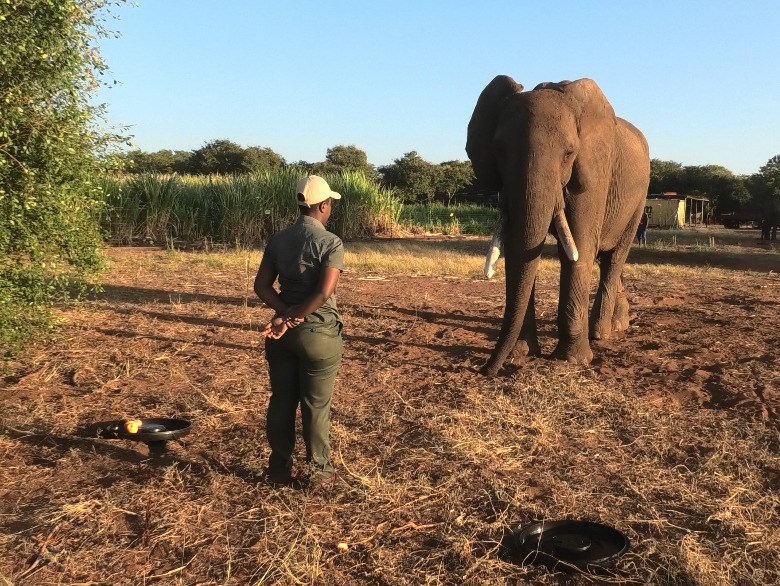
Picture of the experimental set-up. The experimenter (Y.N.M.) waits facing the subject Doma during the experimental trial.

Trials were conducted at the end of the elephants’ daily training sessions. Experimenters were two people familiar to the subjects but who were not involved in elephant training, to avoid the subjects conflating the trials with training. One experimenter conducted all trials across all elephants from eleCREW and another one across all elephants from WildHorizons. Following Cartmill & Byrne [19], subjects received one familiarization trial corresponding to the goal met condition. The order of experimental trials was pseudorandomized, and the positioning of the desired and non-desired items (i.e. left or right) was counterbalanced across trials. For each elephant, experimental trials started at least 2 days after the familiarization trial and were conducted with a minimum interval of 1 day between trials for each elephant. During trials, the handlers ensured the other elephants turned to face the opposite direction and engaged with them to prevent elephants from observing, and potentially learning from, the subjects’ gesturing. Video recordings were made with a Panasonic HC-VXF1 from a 30°–45° angle with respect to the position of the elephant to best discriminate the directedness of any gestural actions (see §2.4; figure 1).

### 2.3. Experimental procedure

Before the trials, V.E. positioned the video camera on a tripod, started the recording and hid behind an object or vegetation nearby to be able to signal to the experimenter when the time had elapsed, while avoiding being perceived by the subjects as a potential recipient. Then a helper who was familiar to the elephant positioned a tray with six apples (i.e. desired item) and an empty tray (i.e. non-desired item) 3 m apart and out of reach of the subject, who remained tethered from the earlier daily training session and so was unable to reach the items. Due to empirical evidence that elephants use olfactory information in foraging-related decision-making [46–48] and to ensure that the subjects knew which tray did not contain the apples, the helper picked up the empty tray and allowed the subject to smell it (N.B. here we presented the empty tray instead of the apple tray to avoid teasing the subjects with the apples without allowing them to pick them from the tray). The helper then went next to V.E. hiding behind an object or vegetation and the experimental trials began. The trials consisted of three experimental phases. (i) *Pre-delivery phase*: the experimenter entered the experimental area. To ensure that the subjects perceived the trial as a feeding session, instead of a training session, and were motivated to obtain the desired item, the experimenter picked up one apple from the apple-baited tray and gave it to the subject, repeating this three times. Then the experimenter positioned herself/himself in between the trays and, when the subject finished eating the three bait apples, waited 40 s facing and visually attending to the subject without moving. When the 40 s elapsed, V.E. produced an audible signal (e.g. ‘apples’, ‘empty’ and ‘one apple’) to inform the experimenter on the start of the next phase and remind them what item to deliver according to the condition. The experimenter then brought one of the two trays within the subject’s reach, delivering the items according to the condition: in the goal met condition, the subject was presented with the apple tray and allowed to take the three remaining apples (i.e. desired item); in the goal not met condition, the subject was presented with and allowed to investigate the empty tray (i.e. non-desired item); in the goal partially met condition, the subject was presented with the apple tray and allowed to take one of the three remaining apples from the apple tray, ensuring that two apples were left on the tray (i.e. desired item). (ii) *Post-delivery phase*: after delivery, the experimenter positioned the trays back in their previous location, positioned herself/himself again in the middle of the trays and, when the subject finished eating any apple, waited for another 40 s facing and visually attending to the subject without moving. When the 40 s elapsed, V.E. produced another audible signal (i.e. ‘leave’) to inform the experimenter of the start of the next phase. (iii) *No-experimenter phase*: the experimenter left the experimental area, and the subject remained alone in front of the trays for another 40 s. All trials ended with the helper removing the trays and the experimenter giving some pellets to the subject to mitigate any frustration in conditions where the subjects did not receive all the apples (figure 1 and electronic supplementary material, videos S1 and S2). After the end of the session (lasting around 5−7 min in total), all subjects were untethered, as is usual after training sessions, and allowed to leave the experimental area.

### 2.4. Video coding

Videos were transferred to a MacBook Pro and coded with the video coding software Elan 6.2. We annotated all vocalizations and gestures produced by the subject during trials. Gestures were considered as any conspicuous mechanically ineffective movement of a body part that was not effective at achieving the goal. Specifically, mechanically effective movements, such as grabbing the tray, as well as movements used for locomotion, feeding or self-directed activities, were not considered gestures (following [30,31,45]). Videos were coded at two levels: the experimental trial level and the signal record level (table 2). For the experimental trial level, we annotated information regarding the trial: the trial number, the experimental phase, the experimental condition, the subject and the positioning of the desired item (i.e. left or right). For the signal record level, we annotated information on the gestures or vocalizations produced, for example: the type of signal (table 3), the number of the signal coded and the direction of any gestural action (table 2).

**Table 2.** List of experimental trial and signal record variables coded.

variable	description
experimental trial	the number of the experimental trial being coded
file name	the name of the video file being coded
subject	the subject of the experimental trial (e.g. Doma, Kariba)
experimental phase	the phase of the experimental trial: before item delivery (i.e. pre-delivery), after item delivery (i.e. post-delivery), when the experimenter left the experimental area (i.e. no-experimenter)
experimental condition	the experimental condition (i.e. goal met, goal not met, goal partially met)
desired item position	the position of the desired item (i.e. left or right)
signal record	the gesture or vocalization type produced by the subject (e.g. trunk-reach-experimenter; trunk-swing-experimenter)
signal number	the number of the signal being coded (e.g. VES0001)
gesture directedness	for signals consisting in gestures, marks if the gestural action is directed to the experimenter, to one of the items, towards the subject’s own body, or if it involves no directedness (not directed to the experimenter or item of interest, as in the case of e.g. trunk-raise)
signal analysis	indicates whether the signal should be included in the analysis (i.e. exclude was used if the signal record had its onset beyond 40 s after the arrival of the experimenter in between the trays)
signal comment	any comment of interest for the signal coded

**Table 3.** Definitions of all identified gesture types with total frequencies of production (number of tokens) in all subjects and in males and females separately. Gesture types observed in single individuals are highlighted with asterisks.

gesture type	total frequency	female frequency	male frequency	definition
ear-flapping	2	1	1	flapping the ears forward
head-nodding	2	1	1	nodding the head vertically up and down
present-stick*	3	3	0	grabbing a stick with the trunk and showing it to the experimenter
stick-on-head*	3	3	0	grabbing a stick with the trunk and placing it on the head
stick-on-temporal-gland*	1	0	1	grabbing a stick with the trunk and holding it placed on the temporal gland
stick-on-tusk*	1	1	0	grabbing a stick with the trunk and placing it on the tusk
trunk-8-swing*	1	0	1	swinging the trunk laterally making an 8-shape movement going up and down on each side
trunk-blow	9	1	8	blowing air through the trunk
trunk-blow-experimenter	3	2	1	blowing air through the trunk towards the experimenter
trunk-curled-under-pull-blow*	1	0	1	curling the trunk inward against the front of the body and then pulling it down and blowing through it at the same time
trunk-fling	2	0	2	tossing the trunk in the air with no apparent direction
trunk-fling-blow-experimenter	24	14	10	tossing the trunk forward in the direction of the experimenter and simultaneously blowing through it
trunk-fling-experimenter	35	15	20	tossing the trunk forward in the direction of the experimenter
trunk-fling-object*	1	0	1	tossing the trunk forward in the direction of one tray (N.B. only performed towards the apple tray)
trunk-grab-tusk*	1	1	0	grasping own tusk with the distal portion of the trunk
trunk-hitting-ground-with-stick*	4	0	4	hitting the ground with a stick held by the trunk
trunk-hitting-head*	2	0	2	hitting own head with the trunk
trunk-over-head*	4	0	4	raising the trunk up to place it over the head
trunk-over-head-open-mouth	6	1	5	raising the trunk up to place it over the head and opening the mouth wide
trunk-over-tusk	5	1	4	placing the trunk over a tusk and keeping it still
trunk-raise	30	9	21	raising the trunk above the head
trunk-raise-blow*	1	0	1	raising the trunk above the head and simultaneously blowing through it
trunk-raise-open-mouth	32	26	6	raising the trunk above the head and opening the mouth wide
trunk-raise-rock*	2	0	2	raising a rock in the air with the trunk
trunk-reach-blow-experimenter	13	9	4	reaching the trunk towards the experimenter and simultaneously blowing through it
trunk-reach-experimenter	36	19	17	reaching the trunk towards the experimenter
trunk-reach-object	9	1	8	reaching the trunk towards one of the trays (N.B. only performed towards the apple tray)
trunk-side-swing*	2	0	2	swinging the trunk from side to side
trunk-swing-blow-experimenter	14	2	12	swinging the trunk back and forth towards the experimenter while simultaneously blowing through it
trunk-swing-experimenter	14	4	10	swinging the trunk back and forth towards the experimenter
trunk-swing-object	2	2	0	swinging the trunk back and forth towards one of the trays (N.B. only performed towards the tray with apples)
trunk-swing-touch-body*	5	5	0	swinging the trunk back and forth leading to purposefully touch own body
trunk-throw-sand*	3	0	3	grabbing sand with trunk and forcefully throwing it in the air with no apparent direction
trunk-throw-sand-experimenter	14	0	14	grabbing sand with trunk and forcefully throwing it towards the experimenter
trunk-throw-sand-self	22	0	22	grabbing sand with trunk and forcefully throwing it on own head. N.B. cases of slight dusting behaviour were not considered as they could have represented self-directed activities
trunk-throw-stick*	1	0	1	grabbing a stick with trunk and throwing it in the air
trunk-throw-stick-experimenter*	1	0	1	grabbing a stick with trunk and throwing it towards the experimenter
trunk-tuck-tusk*	2	0	2	tucking the distal portion of the trunk in between the base of the trunk and a tusk

### 2.5. Statistical analyses

For statistical analyses, we focused on gestures, as vocalizations were only occasionally produced by only four subjects. For consistency across trials, we included gestures whose onset fell within 40 s from when the experimenter reached their position in the middle of the items in both pre-delivery and post-delivery phases and within 40 s from when the experimenter left the experimental area in the no-experimenter phase. We excluded cases where it was unknown or unclear whether a body movement was a gesture. For example, we included only trunk-reach actions that were clearly directed at the experimenter or at the items. Head-shake actions were excluded as they have previously been described as a non-directed expression of annoyance in elephants [42] and because our elephants mostly produced these while turning away from the experimental set-up when experimenters were not responding. We excluded *n* = 11 body acts that could have potentially represented self-directed activities (e.g. leaning towards the experimenter or inconspicuously touching an own body part).

We defined a ‘gesture token’ as each instance of use of a gesture. For example, a communication including a trunk-reach followed by two trunk-fling gestures consists of three gesture tokens (trunk-reach, trunk-fling and trunk-fling) and two gesture types (trunk-reach and trunk-fling). We used a series of generalized linear mixed models (GLMM) to test our predictions. The persistence model tested for persistence by comparing the number of gesture tokens used before (pre-delivery) and after item delivery (post-delivery) across the three experimental conditions. We included the number of gesture tokens (i.e. Gesture_Record_Count) as the response variable. The response was overdispersed so we used a GLMM with a negative binomial distribution to control for overdispersion [49]. We included the interaction between the delivery phase and the experimental condition as the predictor. The sample size consisted of *n* = 313 gesture tokens produced by *n* = 17 subjects across a total of *n* = 51 experimental trials and *n* = 102 pre-delivery and post-delivery phases.

We considered as ‘novel’ any gesture type that had not been used by the subject during pre-delivery and as ‘repeated’ any gesture type that had been used by the subject during pre-delivery. The elaboration model tested for elaboration by comparing the frequency of use of novel as compared with repeated gesture types across the three experimental conditions during post-delivery. To do so, in our dataset, we included a categorical variable to distinguish between novel and repeated gesture types (i.e. Nov_Rep_Type with categories ‘Novel’ and ‘Repeated’). Then, for each experimental trial, we counted the number of times subjects produced, respectively, novel or repeated gesture types under the variable Gesture_Record_Count. This resulted in each experimental trial now having two rows, one reporting the number of gesture tokens that were novel and one the number of gesture tokens that were repeated. For example, if a subject gestured a total of two times during post-delivery, and he did so by only repeating a gesture type he had used during pre-delivery, he would be considered using 2 ‘repeated’ gesture tokens and 0 ‘novel’ gesture tokens (see elaboration model in ‘Experiment-analysis.rmd’ for data structure). We then included in our model Gesture_Record_Count as the response variable and the interaction between the experimental condition (i.e. Experimental_Condition) and Nov_Rep_Type as the predictor. We used a zero-inflated negative binomial GLMM because the response variable was overdispersed and zero inflated [50]. We removed three trials where no gesture tokens were used at all during pre-delivery and post-delivery and another 11 trials where no gesture tokens were used at all during post-delivery. The final sample size consisted of *n* = 121 gesture tokens produced by *n* = 15 subjects across *n* = 37 experimental trials and *n* = 37 post-delivery phases.

The diversity model tested for diversity by comparing the number of different gesture types used before and after delivery (pre-delivery and post-delivery phases) in the three experimental conditions. We included the number of different gesture types (i.e. Gesture_Type_Count) as the response variable. The response was Poisson distributed, so we used a GLMM with a Poisson distribution [49]. We included the interaction between the delivery phase and the experimental condition as the predictor. We controlled for the number of gesture tokens (i.e. Gesture_Record_Count) to account for the increased opportunity to produce more gesture types where the subject produced a larger number of gesture tokens. The sample size consisted of *n* = 197 gesture tokens that were of different types produced by *n* = 17 subjects across a total of *n* = 51 experimental trials and *n* = 102 pre-delivery and post-delivery phases. In all models, because the samples were composed of gesture tokens collected from the same subjects, we included the subject as a random effect. We also controlled for sex and age group (i.e. age; table 1) by including them as control fixed effects. The no-experimenter phase was not used to statistically test for audience directedness because only one of the 17 subjects (Detema) gestured once in this experimental phase (see §3.1).

For all models, to explore the effect of the predictors, we used a likelihood ratio test comparing the full model with a reduced model without the predictors but including the control fixed effects and the random effect [51]. We conducted Tukey post hoc tests to verify that the pre-delivery phases did not differ across conditions (as expected) and Sidak post hoc tests to compare the levels of the interaction terms against each other, according to our predictions (see predictions at the end of §1, [52]). Specifically, for the persistence model, we tested for increased persistence when the goal was not fully met by comparing both the levels goal not met and goal partially met against the level goal met. For the elaboration model, we tested for higher use of novel gesture types when the goal was not met by comparing the level goal not met against both the levels goal met and goal partially met. Lastly, for the diversity model, to test for higher use of gesture types when the goal was not met, we compared the level goal not met against both the levels goal met and goal partially met. We checked for multicollinearity among predictors using variance inflation factors (VIF; [53]). All models had fixed effects with VIFs close to 1.0, indicating no issues of multicollinearity. We assessed model stability by comparing the full model estimates with estimates from models where the levels of the random effect (subject) were removed one at a time [54]. The persistence model was slightly unstable for the condition goal not met (table 4). The elaboration model was slightly unstable for the control fixed effect age. The diversity model was very unstable for different variables (see electronic supplementary material, table S1), so we chose not to interpret the results but provided them in the electronic supplementary material for reference (see electronic supplementary material, diversity model results). We fitted all models using the statistical software R v. 4.0.2 with the packages *lme4* v. 1.1-23 and *glmmTMB* v. 1.1.9 [50,55,56]. We computed post hoc tests with the package *emmeans* v. 1.10.2 [57] and effect sizes for all models using the function *r.squaredGLMM* of the package *MuMIn* v. 1.43.17 [58]. We present the 95% confidence intervals.

**Table 4.** Results of the persistence model. The model tested for the effect of the interaction between the delivery phase and the experimental condition on the total number of gesture tokens produced by the subjects. *n* = 313 gesture tokens by *n* = 17 subjects across a total of *n* = 51 experimental trials and *n* = 102 pre-delivery and post-delivery phases. The table shows estimates, standard errors, *z* values, *p* values, bootstrapped confidence intervals and minimum and maximum of the model stability estimates after removing the levels of the random effect (i.e. subject) one at a time. Relevant significant results (the interactions) are highlighted in bold. ‘(1)’ Not indicated because of limited interpretation.

	estimate	s.e.	z value	*p*	lwr CI	upr CI	min	max
(intercept)	1.403	0.264	5.319	(1)	0.858	1.876	1.295	1.545
Experimental_Condition goal not met	−0.141	0.260	−0.542	0.588	−0.686	0.344	−0.363	0.013
Experimental_Condition goal partially met	−0.139	0.258	−0.537	0.591	−0.659	0.369	−0.292	−0.025
Delivery_Phase post-delivery	**−0.973**	**0.296**	**−3.282**	**0.001**	**−1.565**	**−0.413**	**−1.087**	**−0.891**
Age young adult	−0.466	0.213	−2.186	0.029	−0.868	−0.064	−0.574	−0.395
Sex male	0.472	0.208	2.272	0.023	0.082	0.882	0.347	0.546
Experimental_Condition goal not met: Delivery_Phase post-delivery	0.449	0.413	1.088	0.276	−0.304	1.289	0.183	0.615
Experimental_Condition goal partially met: Delivery_Phase post-delivery	**1.045**	**0.397**	**2.632**	**0.008**	**0.282**	**1.865**	**0.817**	**1.166**

Inter-rater reliability was calculated on 10% (i.e. six, including three from eleCREW and three from Wild Horizons) randomly selected experimental trials of the three different conditions coded by the lead author (V.E.) and a trained coder (Anna Letrari). Reliability was assessed at the level of the gesture record type coded, as well as on the number of gesture tokens and the number of gesture types coded in each trial. We found almost perfect reliability on all variables (unweighted Cohen’s kappa for gesture record type: *k* = 0.909, *z* = 13.8, *p* < 0.001; intra-class correlation coefficient for number of gesture tokens: *r* = 0.984, *F* (11,11) = 124, *p* < 0.001; intra-class correlation coefficient for number of gesture types: *r* = 0.850, *F*(11,11) = 12.4, *p* < 0.001).

## 3. Results

We recorded a total of *n* = 313 gesture tokens of *n* = 38 types from *n* = 17 subjects from the three elephant groups, across *n* = 51 experimental trials (table 3). Of the gesture tokens involving a directed action (*n* = 202), *n* = 161 were directed at the experimenter, *n* = 12 at the tray with apples and *n* = 29 at their own body. No gesture appeared to be directed at the empty tray. Out of the *n* = 38 gesture types, *n* = 21 were produced by only one elephant group (of which *n* = 10 types were produced by eleCREW, six by WildHorizons1 and five by WildHorizons2). Out of the *n* = 38 gesture types, *n* = 19 were specific to single individuals (i.e. not observed in at least two individuals).

### 3.1. Audience directedness

No subjects, with the exception of one male for one gesture token (Detema; table 1), gestured when the experimenter was not present (i.e. in the no-experimenter phase), showing that gestures were almost exclusively performed in the presence of an audience (the experimenter, who was always attentive).

### 3.2. Persistence

**Figure 2.**
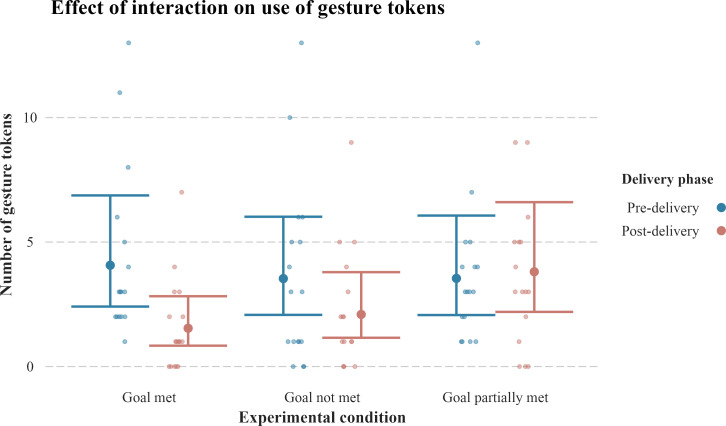
Predicted mean number of gesture tokens used by the subjects before and after item delivery in the three experimental conditions. The large dots with error bars depict the predicted mean values and their confidence intervals. The small dots represent the data points. *n* = 313 gesture tokens by *n* = 17 subjects across a total of *n* = 51 experimental trials and *n* = 102 pre-delivery and post-delivery phases. We found that subjects used more gesture tokens during post-delivery in the goal partially met condition as compared with the goal met condition.

Overall, the interaction between the delivery phase and the experimental condition affected the number of gesture tokens used by the subjects (*χ*^2^_2_ = 6.953, *p =* 0.031). In general, the subjects decreased their use of gesture tokens after item delivery (table 4). As predicted, the subjects increased the number of gesture tokens produced from pre-delivery to post-delivery more in the goal partially met condition as compared with the goal met condition. However, against our predictions, there was no difference in the use of gesture tokens from pre-delivery to post-delivery between the goal not met condition and the goal met condition (figure 2, table 4 and electronic supplementary material, table S2): in both conditions, the subjects decreased their number of gesture tokens from pre-delivery to post-delivery (figure 2). When considering the post-delivery phase only, we similarly found that the subjects produced more gesture tokens in the goal partially met condition as compared with the goal met condition (Tukey post hoc test: estimate = −0.906, s.e. = 0.303, *z* value = −2.986, *p* = 0.008), while again we found no difference between the goal not met and goal met conditions (Tukey post hoc test: estimate = −0.308, s.e. = 0.320, *z* value = −0.963, *p* = 0.600). Pre-delivery, the number of gesture tokens did not vary across conditions (electronic supplementary material, table S3), indicating that the subjects could not tell the experimental condition (i.e. type of item delivery) beforehand. The model explained a moderate proportion of variance (marginal *R*^2^ = 0.30).

### 3.3. Elaboration

**Figure 3.**
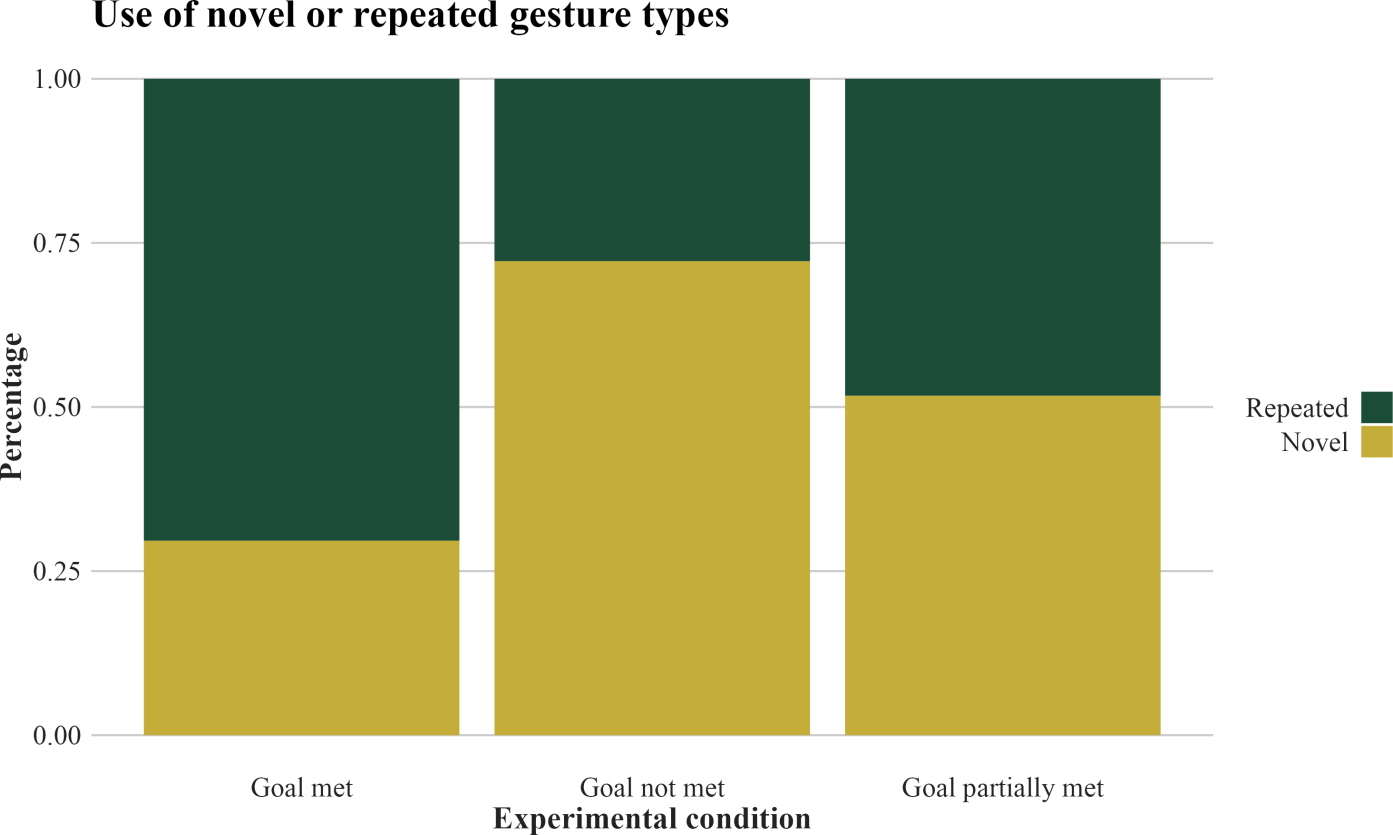
Percentage of novel or repeated gesture types used by the subjects during post-delivery in the three experimental conditions. *n* = 121 gesture tokens produced by *n* = 15 subjects across *n* = 37 experimental trials and *n* = 37 post-delivery phases. We found that, during post-delivery, subjects used more often novel gesture types rather than repeated gesture types (i.e. gesture types already used during pre-delivery) in the goal not met condition as compared with the goal met condition.

Overall, the interaction between the experimental condition and whether the gesture types were novel or repeated (i.e. Nov_Rep_Type) affected the number of novel or repeated gesture tokens used by the subjects during post-delivery (*χ*^2^_2_ = 6.741, *p* = 0.034). As predicted, during post-delivery, the subjects used novel gesture types more often in the goal not met condition than in the goal met condition (figure 3 and table 5). However, we found no difference in the frequency of use of novel gesture types between the goal not met and the goal partially met conditions (Sidak post hoc test: estimate = −0.762, s.e. = 0.600, *z* value = −1.271, *p* = 0.495). The model explained a low proportion of variance (marginal *R*^2^ = 0.212).

**Table 5.** Results of the elaboration model. The model tested for the effect of the interaction between the experimental condition and whether the gesture types were novel or repeated (i.e. Nov_Rep_Type) on the number of novel or repeated gesture tokens produced by the subjects during post-delivery. *n* = 121 gesture tokens produced by *n* = 15 subjects across *n* = 37 experimental trials and *n* = 37 post-delivery phases. The table shows estimates, standard errors, *z* values, *p* values, confidence intervals and minimum and maximum of the model stability estimates after removing the levels of the random effect (i.e. subject) one at a time. Relevant significant results (the interactions) are highlighted in bold. ‘(1)’ Not indicated because of limited interpretation.

	estimate	s.e.	z value	*p*	lwr CI	upr CI	min	max
(intercept)	0.308	0.392	0.785	(1)	−0.461	1.076	−0.070	0.428
Experimental_Condition goal not met	−0.663	0.478	−1.387	0.165	−1.600	0.274	−1.011	−0.220
Experimental_Condition goal partially met	0.286	0.402	0.710	0.477	−0.502	1.073	0.066	0.568
Nov_Rep_Type novel	−0.854	0.504	−1.695	0.090	−1.841	0.133	−1.314	−0.421
Age young adult	−0.068	0.275	−0.247	0.805	−0.606	0.470	−0.221	0.042
Sex male	0.335	0.276	1.214	0.225	−0.206	0.875	0.261	0.410
Experimental_Condition goal not met: Nov_Rep_Type novel	**1.775**	**0.685**	**2.592**	**0.010**	**0.433**	**3.118**	**1.150**	**2.340**
Experimental_Condition goal partially met: Nov_Rep_Type novel	1.013	0.629	1.611	0.107	−0.220	2.246	0.625	1.463

## 4. Discussion

Here we investigated whether elephants gesture intentionally by testing for audience directedness, persistence and elaboration. We show that semi-captive elephants gesture in the presence of a visually attentive audience, that they persist in further gesturing when their goal was partially met, as compared with fully met, and that they elaborate their gesturing after previous communicative attempts failed to meet their goal.

Previous studies have shown that apes (including human infants) use gestures in the presence of an attentive audience [7,59–62]. In our study, 16 of the 17 subjects gestured only when a visually attentive experimenter was present. A previous study, also conducted at WildHorizons, showed that these semi-captive elephants produced more visual gestures when the experimenter was present and facing them as compared with when the experimenter was absent or facing away from them [44]. By showing here that our subjects almost exclusively gestured when the experimenter was present and attentive, our results strongly support earlier findings that elephants selectively employ visual gestures in the presence of a visually attentive audience. Moreover, our previous study, also conducted at eleCREW, demonstrated that semi-captive elephants appropriately select the sensory modality of their gestures based on the visual attention of a conspecific audience [45]. Specifically, elephants used more silent-visual gestures when conspecifics were visually attending and more tactile ones when conspecifics were not visually attending to them. Sensitivity to others’ visual attentiveness has also been found in apes and in species like dogs, pigs and scrub jays [7,30,63–66]. Future studies should explore audience directedness in free-ranging elephant gestural communication and investigate whether elephants habituated to human presence appropriately adjust the sensory modality of their gestures when communicating towards visually inattentive humans.

We also found that elephants directed more than half of their gestures towards the experimenter and some towards the tray with apples, while they directed no gestures towards the empty tray, suggesting the latter was of no interest. These findings support the inference that their communicative attempts were *about* getting the apples (i.e. they were goal-directed; [8]). While we remain agnostic on whether the gestures directed at the apples represent cases of pointing to requests for the apples, we invite future studies to explore referential communication in elephants, a topic that remains under-explored despite showing promise [67]. For example, presenting semi-captive elephants with a preferred and a non-preferred food item might elicit more explicit referential communication in order to disambiguate their preference, as shown in captive chimpanzees [8].

Previous studies found that great apes persist in gesturing to both human and conspecific recipients when they do not meet their goals [7,8,11,19,68]. Specifically, the two experimental studies on which our study was based [8,19] showed that captive chimpanzees and orangutans persist in further gesturing when their goals were not met or only partially met, as compared with when they were met. We found mixed results in gesture persistence with our subjects. The semi-captive elephants increased their use of gesture tokens (i.e. persisted more) from the pre-delivery to post-delivery phase, and overall used more gesture tokens during post-delivery, in the goal partially met condition as compared with the goal met condition. However, in both the conditions goal not met and goal met, our subjects decreased their use of gesture tokens during the post-delivery phase, and there was no significant difference in the degree of persistence between these two conditions (figure 2).

There are various possible interpretations for these results. The semi-captive elephants in this study differ from the captive chimpanzees and orangutans of the earlier studies, in that the elephants undergo positively reinforced behavioural training to respond to verbal or visual cues given by the handlers [69]. Compared with the captive apes in Leavens *et al.* [8] and Cartmill & Byrne [19], in our experimental procedure, the subjects initially received three apples so that they recognized the trial as a feeding, rather than a training session. The subjects may have thus considered delivery of the empty tray in the goal not met condition as the ‘end’ of the feeding session, leading them to gesture less than during pre-delivery. The elephants could have also been considering the experimenter’s motivation to give them the apples. Elephants are trained so that handlers give elephants food pellets as rewards when they respond with the correct behaviour (e.g. enter the stable, follow them). Handlers keep pellets in a pouch tied to their belts. Elephants can smell the pellets and often beg for them by reaching or cupping their trunks up towards the handlers or the pouches (V.E., personal observation). To avoid continuous requests by the elephants, handlers usually do not respond to such communicative attempts and command the elephants to stop begging. Given these factors, our subjects may have interpreted receiving the empty tray as a cue that the experimenter was refusing to provide apples, resulting in more limited persistence in the goal not met condition (as compared with the increase seen in the goal partially met condition, where the experimenter gave them again some apples). Regardless of interpretation, *within* each delivery phase, the elephants used multiple gesture tokens (electronic supplementary material, table S2), showing that they do persist in gesturing when the experimenter is visually attending to them but not reacting, thus meeting the criterion for persistence in goal-directed intentional communication.

In addition, and in contrast to the captive apes who stopped gesturing when experimenters fully met their goal [8,19], the elephants did not completely cease gesturing in the goal met condition after receiving all the available apples. Elephants possess an exceptionally advanced olfactory sense [46,70,71], and the subjects are used to requesting and receiving food that is not visible to them (e.g. pellets in the handlers’ pouches). Although there were no more apples on the apple tray, the subjects may have continued to produce an occasional gesture (electronic supplementary material, table S2), because they were probably able to smell the apples in storage for future trials kept in a closed hut or car around 50 m away.

In our study, elaboration was defined as the use of novel signals following the failure of a previous communication [19]. Specifically, we considered elaboration during post-delivery as the use of *novel* gesture types that had not been used during pre-delivery. Cartmill & Byrne [19] found that captive orangutans used novel gesture types more often after previous gesture types failed at meeting their goal, while repeated the same gesture types after these succeeded at partially meeting their goal. The authors interpreted these findings as suggesting that orangutans evaluate the communicative effectiveness or success of previous communicative attempts when gesturing and as evidence of possible second-order intentionality. Specifically, they considered the fact that orangutans could discriminate between the different types of ‘failure’ (i.e. goal not met, goal partially met) as indicative of them taking into account different degrees of understanding by their recipient. Delivery of the non-desired food suggested misunderstanding of the goal—and resulted in the use of novel gesture types. Delivery of part of the desired food suggested that the goal was understood, but not completed—and resulted in repeated use of the previously used gesture types.

We similarly found that elephants used more novel gesture types, rather than repetitions of previous gesture types, after the experimenters did not meet their goal as compared with when they did meet their goal, showing elaboration. Remaining conservative in terms of second-order intentionality, we interpret our results as indicating that elephants, like orangutans, at least take into account the effectiveness of their previous communicative attempts when gesturing. In addition, because the elephants continued to gesture following the goal met condition (perhaps, as mentioned above, because they were aware of more apples in the vicinity), we could also show that they repeated gesture types previously used when their goal had been met, as compared with not met (figure 3), which suggests that they understand that previous gesture types had been successful and thus repeat them. However, we found no statistical difference in the use of novel gesture types (as compared with repeated gesture types) between the goal not met and the goal partially met conditions. Taken together, these results suggest that elephants may be more motivated to use gestural elaboration after complete failure of previous communication rather than use gestural repetition after partial success. Future studies should further investigate the use of gestural elaboration versus repetition, to assess second-order intentionality in elephant communication.

## 5. Conclusion

Goal-directed intentionality is a core feature of human language that enables us to use large repertoires of signals to express a wide range of meanings and serves as a fundamental prerequisite for second-order intentionality [3,6]. There is abundant evidence that all non-human apes gesture with goal-directed intentionality using very large sets of gesture types and that they do so flexibly towards a diverse set of social goals (i.e. meanings, [7]). In contrast, evidence in other species, including non-anthropoid primates [14,15,72], is scarce and, in non-primates, typically restricted to a few highly specific signals towards fixed goals [20–22]. We provide the first evidence that semi-captive elephants use many gesture types towards a visually attentive audience, that they persist in gesturing when they do not fully meet their goal and that they elaborate their gesturing following the failure of previous communicative attempts, when communicating to ask for food. Together, this pattern of results provides the first systematic evidence of goal-directed intentionality across many gesture types in elephant communication and in non-primate. Since the ability to flexibly communicate intentionally using many signal types across diverse goals characterizes human language and ape gesture [3,7,12,28], future studies could apply our experimental design across different goals to test for flexible intentional gesturing in semi-captive elephants. Elephants last shared a common ancestor with humans around 100 million years ago [73]. But, like humans and other apes, elephants live in a multi-level fission–fusion social system and possess sophisticated cognition [74,75]. Elephants form diverse and long-term relationships with different individuals [76–78] and may benefit from a large, flexible system of communication in which they can use various gesture types to intentionally communicate diverse behavioural and social goals. Our findings suggest that goal-directed intentional gestural communication emerged through convergent evolution in distant taxa (including elephants and apes) facing similar socio-cognitive pressures. Future studies should explore whether free-ranging elephants gesture intentionally to conspecifics and describe the repertoire, meanings and flexible use across goals and contexts of elephant gestures. Finally, we encourage future research to explore intentionality in other highly social species that employ large sets of signals in order to further our understanding of the convergent evolution of this capacity.

## Data Availability

The data and code for this study can be accessed in this Zenodo repository [79]. Supplementary material is available online [80].
